# Effects of Dopamine D_2_ Receptor Partial Agonist Antipsychotic Aripiprazole on Dopamine Synthesis in Human Brain Measured by PET with L-[β-^11^C]DOPA

**DOI:** 10.1371/journal.pone.0046488

**Published:** 2012-09-28

**Authors:** Hiroshi Ito, Harumasa Takano, Ryosuke Arakawa, Hidehiko Takahashi, Fumitoshi Kodaka, Keisuke Takahata, Tsuyoshi Nogami, Masayuki Suzuki, Tetsuya Suhara

**Affiliations:** Molecular Imaging Center, National Institute of Radiological Sciences, Chiba, Japan; Chiba University Center for Forensic Mental Health, Japan

## Abstract

Dopamine D_2_ receptor partial agonist antipsychotic drugs can modulate dopaminergic neurotransmission as functional agonists or functional antagonists. The effects of antipsychotics on presynaptic dopaminergic functions, such as dopamine synthesis capacity, might also be related to their therapeutic efficacy. Positron emission tomography (PET) was used to examine the effects of the partial agonist antipsychotic drug aripiprazole on presynaptic dopamine synthesis in relation to dopamine D_2_ receptor occupancy and the resulting changes in dopamine synthesis capacity in healthy men. On separate days, PET studies with [^11^C]raclopride and L-[β-^11^C]DOPA were performed under resting condition and with single doses of aripiprazole given orally. Occupancy of dopamine D_2_ receptors corresponded to the doses of aripiprazole, but the changes in dopamine synthesis capacity were not significant, nor was the relation between dopamine D_2_ receptor occupancy and these changes. A significant negative correlation was observed between baseline dopamine synthesis capacity and changes in dopamine synthesis capacity by aripiprazole, indicating that this antipsychotic appears to stabilize dopamine synthesis capacity. The therapeutic effects of aripiprazole in schizophrenia might be related to such stabilizing effects on dopaminergic neurotransmission responsivity.

## Introduction

Effects of antipsychotic drugs with antagonistic property mediated by blockade of postsynaptic dopamine D_2_ receptors can be evaluated by positron emission tomography (PET) studies for determining the occupancy of dopamine D_2_ receptors in schizophrenia patients treated with first-generation antipsychotics, e.g., haloperidol [1], [2] and second-generation antipsychotics, e.g., risperidone [3], antagonists of dopamine D_2_ receptors. Recently, a new atypical antipsychotic drug acting as a partial agonist of dopamine D_2_ receptors, aripiprazole, has been widely used for the treatment of schizophrenia [4]. Partial agonists of dopamine D_2_ receptors can modulate the dopaminergic neurotransmission as functional agonists or functional antagonists [5].

Effects of antipsychotics on presynaptic dopaminergic functions, e.g., dopamine synthesis capacity, might also be related to their therapeutic effects. The regional activity of aromatic L-amino acid decarboxylase (AADC) in brain, indicating dopamine synthesis capacity, can be estimated using radiolabeled L-DOPA [6]. Animal studies showed significant increases and decreases in dopamine synthesis capacities by antagonists and agonists of dopamine D_2_ receptors using [^3^H]DOPA, L-[β-^11^C]DOPA, and 6-[^18^F]fluoro-L-DOPA, respectively [7]–[9]. These findings suggest that changes in presynaptic dopamine synthesis capacity might be caused by the pharmacological effects on dopaminergic autoreceptors [10]. On the other hand, an increase in dopamine synthesis capacity by administration of the partial agonist antipsychotic aripiprazole was observed in animal studies by measuring DOPA accumulation [11].

Effects of antipsychotics with antagonistic property on dopamine synthesis capacity have been studied in brains of human subjects. The acute administration of the antipsychotic drug haloperidol and the use of PET with 6-[^18^F]fluoro-L-DOPA revealed a significant increase in dopamine synthesis capacity in healthy human subjects [12]. In contrast, in schizophrenia patients, a significant decrease in dopamine synthesis capacity after chronic administration of haloperidol was observed with PET and 6-[^18^F]fluoro-L-DOPA [13]. Recently, we found that the antipsychotic drug risperidone could be considered to stabilize dopamine synthesis capacity in healthy human subjects, indicating that the therapeutic effects of risperidone in schizophrenia might be related to the stabilizing effects on dopaminergic neurotransmission responsivity [14]. However, the effects of the partial agonist antipsychotic aripiprazole on dopamine synthesis capacity have not yet been investigated in human subjects.

In the present study, dopamine D_2_ receptor bindings and dopamine synthesis capacities at resting condition and after oral administration of a single dose of aripiprazole were measured in the same human subjects by PET with [^11^C]raclopride and L-[β-^11^C]DOPA, respectively, to determine changes in dopamine synthesis capacity by this antipsychotic in relation to the occupancy of dopamine D_2_ receptors. Similar experimental protocol as previous our work with risperidone was used, and results were compared [14].

## Results

**Figure 1:**
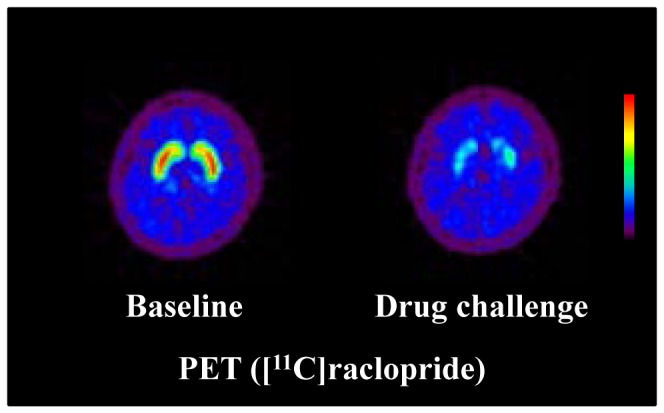
Typical PET summation images of frames between 32–60 min after intravenous injection of [^11^C]raclopride for baseline and drug challenge (6 mg of aripiprazole) studies. The sections are transaxial at the level of putamen.

The occupancies of dopamine D_2_ receptors for each dose of aripiprazole as measured by PET with [^11^C]raclopride ranged from 53% to 79% in the caudate and from 51% to 77% in the putamen (Table 1). Typical images of [^11^C]raclopride for baseline and drug challenge studies are shown in Fig. 1. Reduced uptake of [^11^C]raclopride in the striatum was observed after oral administration of aripiprazole.

**Table 1:** Dose of aripiprazole and ranges of occupancy of dopamine D_2_ receptors.

Dose of aripiprazole	Occupancy (%)	
(mg)	Caudate	Putamen
3	53–61%	51–58%
	(57±4%)	(55±2%)
6	70–77%	66–72%
	(73±3%)	(69±3%)
9	77–79%	75–77%

(mean ± SD).

The plasma concentrations of aripiprazole during [^11^C]raclopride and L-[β-^11^C]DOPA PET studies, averaged between the start and end of each scanning, were 12.0±2.1 ng/mL (mean ± SD) and 10.4±1.5 ng/mL for 3 mg of oral administration dose of aripiprazole, 29.0±2.1 ng/mL and 25.6±2.1 ng/mL for 6 mg, and 39.6–40.4 ng/mL and 38.2–39.7 ng/mL for 9 mg, respectively. The plasma concentrations of dehydroaripiprazole during [^11^C]raclopride and L-[β-^11^C]DOPA PET studies were 0.4±0.2 ng/mL (mean ± SD) and 0.5±0.2 ng/mL for 3 mg of oral administration dose of aripiprazole, 0.9±0.3 ng/mL and 1.1±0.4 ng/mL for 6 mg, and 1.1–1.6 ng/mL and 1.4–2.4 ng/mL for 9 mg, respectively.

**Figure 2:**
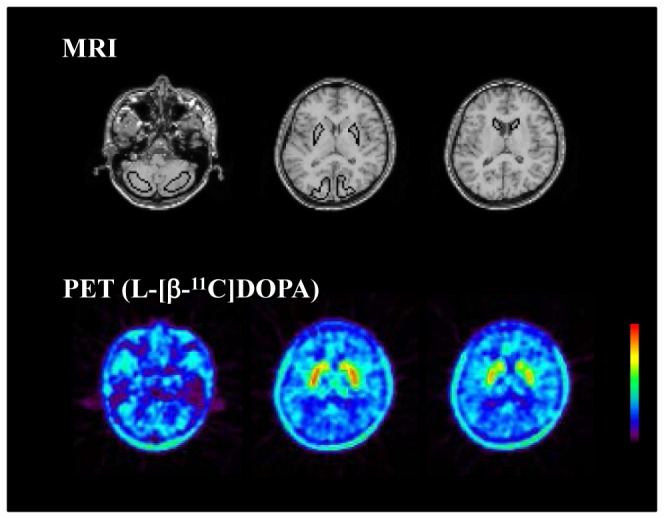
Regions of interest (ROIs) drawn on coregistered MR images. ROIs are defined for the cerebellar cortex, putamen, caudate head, and occipital cortex. Typical PET summation images of frames between 29–89 min after intravenous injection of L-[β-^11^C]DOPA for baseline study are also shown.

The uptake rate constants k_i_ of L-[β-^11^C]DOPA in the caudate and putamen, indicating the dopamine synthesis capacity for baseline and drug challenge studies, are shown in Table 2. No significant differences in k_i_ were observed between the two studies (paired t-test). Typical images of L-[β-^11^C]DOPA for baseline study are shown in Fig. 2. Weighted sums of the natural neutral amino acids (NAAs) concentrations in plasma were 1170±142 nmol/mL for the baseline study and 1122±154 nmol/mL (mean ± SD) for the drug challenge study. The values showed no significant differences between the two studies (paired t-test).

**Table 2:** Dopamine synthesis capacity k_i_ of both baseline and drug challenge studies.

	Caudate	Putamen
Baseline	0.0114±0.0022	0.0134±0.0014
Drug challenge	0.0111±0.0016	0.0136±0.0014

Values are mean ± SD.

Unit is min^−1^.

No significant differences in k_i_ are observed between the two studies (paired t-test).

**Figure 3:**
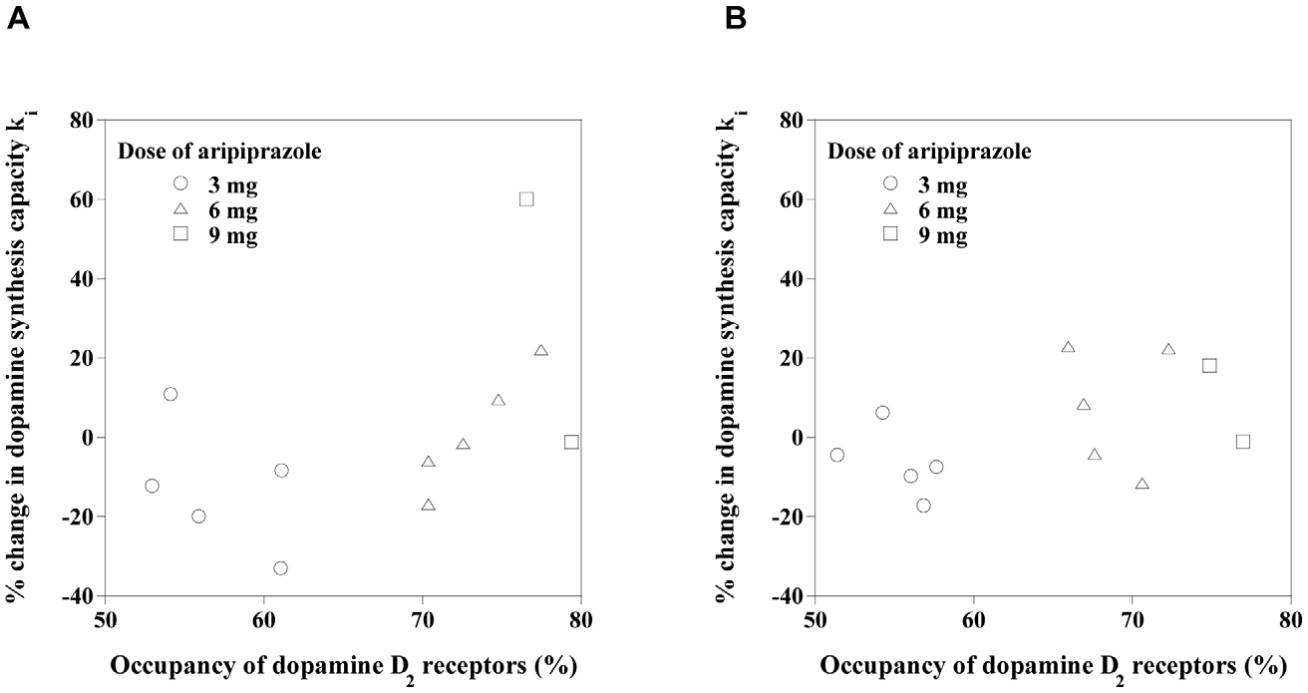
Relations between the occupancy of dopamine D_2_ receptors and the percentage change in k_i_ by drug challenge with aripiprazole in the caudate (A) and putamen (B).

**Figure 4:**
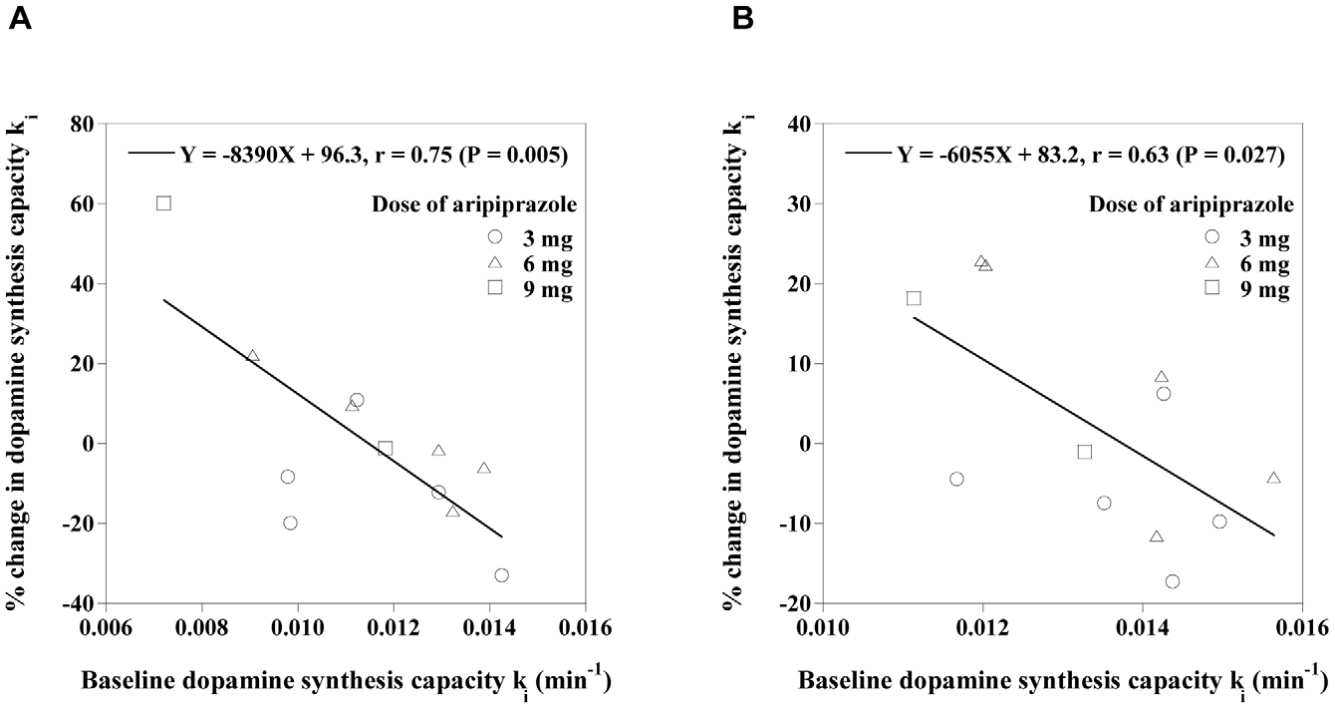
Relations between k_i_ in the baseline study and the percentage changes in k_i_ by drug challenge with aripiprazole in the caudate (A) and putamen (B).

Fig. 3 shows the relations between dopamine D_2_ receptor occupancy and percentage changes in k_i_ by the drug challenge. There were no significant correlations. No dose dependency was observed in percentage changes in k_i_ by the drug challenge. The relations between k_i_ in the baseline study and percentage change in k_i_ by the drug challenge for each administration dose of aripiprazole are shown in Fig. 4. Significant negative correlations were observed among all administration dose (caudate: *P* = 0.005, putamen: *P* = 0.027).

## Discussion

The present study was performed using similar experimental protocol as previous our work with the antipsychotic risperidone, an antagonist for dopamine D_2_ receptors [14]. The effects of antipsychotics on presynaptic dopamine synthesis might be due to pharmacological action on dopaminergic autoreceptors [10] and by neural network regulation. While occupancy of dopamine D_2_ receptors corresponding to the dose of aripiprazole was observed [15]–[18], the current study showed no significant changes in dopamine synthesis capacity by the administration of aripiprazole. There were also no significant correlations between the occupancy of dopamine D_2_ receptors and changes in dopamine synthesis capacity by aripiprazole. These findings are similar to our previous observation in healthy human subjects using risperidone [14]. To our knowledge, this is the first study to investigate the effects of aripiprazole on dopamine synthesis capacity in humans using PET. Significant increases and decreases in dopamine synthesis capacities by antagonists and agonists, respectively, of dopamine D_2_ receptors were observed in animal studies [7]–[9], indicating that pharmacological effects on dopaminergic autoreceptors and the neural network might cause changes in presynaptic dopamine synthesis capacity [10]. An increase in dopamine synthesis capacity by aripiprazole was observed in animal studies [11], although partial agonists for dopamine D_2_ receptors might reduce presynaptic activity through feedback regulation [5], [19]. However, no significant changes in dopamine synthesis capacity by a single administration of an antagonist or a partial agonist were observed in healthy human subjects. The inconsistency of changes in dopamine synthesis capacity between the present study and previous animal studies might be due to differences in administration dose and way of aripiprazole.

In the present study, significant negative correlations were observed between baseline dopamine synthesis capacity and the percentage changes in dopamine synthesis capacity by aripiprazole. This indicates that aripiprazole administration causes either increase or decrease in dopamine synthesis capacity in subjects with low or high baseline dopamine synthesis capacity, respectively, and the degrees of increase and decrease in dopamine synthesis capacity depend on the baseline dopamine synthesis capacities. These findings are similar to our previous observation in healthy human subjects using the antagonist antipsychotic risperidone [14] and a previous report using the antagonist antipsychotic haloperidol [20]. In addition, the coefficients of variation of dopamine synthesis capacity were smaller in studies with the administration of aripiprazole than in baseline studies, the same as with risperidone [14]. These results indicate that the partial agonist antipsychotic aripiprazole can be assumed to stabilize dopamine synthesis capacity in the same way as antipsychotic drugs with antagonistic property. These also indicate that there are two groups in the healthy subjects with relatively high and low baseline dopamine synthesis capacities, however, we could not find any differences between the two groups. Although stabilizing effect of antipsychotic drugs on dopamine synthesis capacity were observed both in the antagonist and partial agonist antipsychotic drugs, its mechanism would be unknown. An abnormal responsivity in both phasic and tonic dopamine release, which might be related to the modulation of dopaminergic neurotransmission, has been considered in the pathophysiology of schizophrenia [21]. The therapeutic effects of aripiprazole might be related to stabilizing effects on such dopaminergic responsivity. It has also been reported that aripiprazole suppressed phasic dopamine release in methamphetamine-sensitized rat [22]. Although the occupancy of dopamine D_2_ receptors ranged from about 50% to 80% in the present study, there might be some kind of threshold of occupancy by aripiprazole for the stabilizing effect of dopamine synthesis capacity to emerge. Further investigations about such threshold should be considered.

The occupancy of dopamine D_2_ receptors in this study might be relatively lower than in previous reports regarding drug challenge studies being performed after daily administration of aripiprazole for more than ten days [15], [18]. Because only an acute intervention was performed in the present study, the occupancy might actually be relatively lower. Aripiprazole treatment has been shown to be well tolerated with a dose up to 30 mg/day [23], and the optimal dose was reported to be 10 mg/day [4]. The doses of aripiprazole administered in this study (3–9 mg) were smaller than those doses. Since the starting dose of aripiprazole was set at 6–12 mg/day in Japan, from an ethical standpoint, a relatively small dose was used in the present study [24]. However, the chronic effects of relatively large doses of aripiprazole on dopamine synthesis capacity should be investigated in patients with schizophrenia in the future. In addition, the relation between changes in dopamine synthesis capacity and changes in clinical symptoms should also be investigated to confirm meaning of stabilizing effects of aripiprazole on dopamine synthesis capacity.

Aripiprazole also has an antagonistic action on serotonin 5-HT_2A_ receptors and a partial agonistic action on 5-HT_1A_ receptors with relatively high affinity [5]. The 5-HT_2A_ receptor antagonists have been reported to modulate endogenous dopamine release [25], and to reduce extrapyramidal side effects [26]–[28]. Since aripiprazole has an antagonistic action on 5-HT_2A_ receptors, it may modulate endogenous dopamine release. These reports suggest that changes in dopamine synthesis capacity by the administration of aripiprazole might be due not only to pharmacological effects on dopaminergic autoreceptors, but also on serotonin 5-HT_2A_ receptors similar to our previous report on risperidone [14]. To clarify this, additional studies using the same design and a selective antagonist for dopamine D_2_ receptors, such as sulpiride, should be performed [14].

In conclusion, dopamine D_2_ receptor bindings and dopamine synthesis capacities at resting condition and after oral administration of a single dose of the partial agonist antipsychotic aripiprazole were measured in the same human subjects. While dose-corresponding occupancy of dopamine D_2_ receptors was observed, no significant changes in dopamine synthesis capacity by aripiprazole administration were observed. In addition, no significant correlation between occupancy of dopamine D_2_ receptors and changes in dopamine synthesis capacity by aripiprazole was observed. On the other hand, a significant negative correlation was observed between baseline and aripiprazole-induced changes in dopamine synthesis capacities, indicating that the partial agonist antipsychotic aripiprazole can be considered as having a stabilizing effect on dopamine synthesis capacity, the same as antagonist antipsychotic drugs. This suggests that the therapeutic effects of aripiprazole in schizophrenia are possibly related to the stabilizing effects on dopaminergic neurotransmission responsivity.

## Methods

### Subjects

The study was approved by the Ethics and Radiation Safety Committees of the National Institute of Radiological Sciences, Chiba, Japan. Twelve healthy men (23–34 years of age, 24.1±3.2 years [mean ± SD]) were recruited and written informed consent was obtained. The subjects were free of somatic, neurological and psychiatric disorders according to their medical history and magnetic resonance (MR) imaging of the brain. No histories of current or previous drug abuse were revealed by interviews.

### PET procedures

All PET studies were performed with a Siemens ECAT Exact HR+ system, providing 63 sections with an axial field of view of 15.5 cm [29]. Intrinsic spatial resolution was 4.3 mm in-plane and 4.2 mm full-width at half maximum (FWHM) axially. With a Hanning filter (cutoff frequency: 0.4 cycle/pixel), the reconstructed in-plane resolution was 7.5 mm FWHM. Data were acquired in three-dimensional mode. Scatter was corrected by a single scatter simulation technique [30]. A 10-min transmission scan using a ^68^Ge-^68^Ga line source was performed for attenuation correction. A head fixation device with thermoplastic attachments for individual fit was used to minimize head movement during the PET measurements.

PET studies were performed under resting condition (baseline study) and oral administration of aripiprazole (drug challenge study) on separate days. The interval between the 2 studies was 7 days in 7 subjects, and 14 days in 5 subjects. In each study, both PET scans with [^11^C]raclopride and L-[β-^11^C]DOPA were performed sequentially. Dynamic PET scanning was performed for 60 minutes following an intravenous rapid bolus injection of [^11^C]raclopride. Then, one hour later, dynamic PET scanning was performed for 89 minutes after intravenous rapid bolus injection of L-[β-^11^C]DOPA. The frame sequence consisted of twelve 20-sec frames, sixteen 1-min frames, and ten 4-min frames for [^11^C]raclopride, and seven 1-min frames, five 2-min frames, four 3-min frames, and twelve 5-min frames for L-[β-^11^C]DOPA. The radioactivity injected was 218–237 MBq and 364–392 MBq in the baseline studies, and 199–233 MBq and 364–415 MBq in the drug challenge studies for [^11^C]raclopride and L-[β-^11^C]DOPA, respectively. Specific radioactivity was 162–239 GBq/µmol and 24–124 GBq/µmol in the baseline studies, and 125–253 GBq/µmol and 17–273 GBq/µmol in the drug challenge studies for [^11^C]raclopride and L-[β-^11^C]DOPA, respectively. A venous blood sample was taken at the beginning of L-[β-^11^C]DOPA PET scanning to measure natural neutral amino acid (NAA) concentration in plasma. The NAA concentration was measured by HPLC (L-8500 amino acid analyzer system, Hitachi Corp., Tokyo, Japan). The amino acids are phenylalanine, tryptophan, leucine, methionine, isoleucine, tyrosine, histidine, valine and threonine, which are transported via the same carrier at the blood-brain barrier as L-DOPA [31]. The weighted sum of the NAAs, which was the L-DOPA corresponding concentration of the nine NAAs for the carrier system, was calculated according to our previous work [32].

In the drug challenge studies, aripiprazole at 3–9 mg was orally administered 3.5 hours before the start of PET scanning with [^11^C]raclopride. The aripiprazole dose was 3 mg in 5 subjects, 6 mg in 5 subjects, and 9 mg in 2 subjects. To estimate the plasma concentration of aripiprazole and its active metabolite, dehydroaripiprazole, venous blood sampling was performed at the start and end of each PET scan [33]. The plasma concentrations of aripiprazole and dehydroaripiprazole, which showed partial agonist effects similar to those of aripiprazole, were determined by the method of validated liquid chromatography coupled to mass spectrometry/mass spectrometry (LC-MS/MS) [34].

All MR imaging studies were performed with a 1.5-Tesla MR scanner (Philips Medical Systems, Best, The Netherlands). Three-dimensional volumetric acquisition of a T1-weighted gradient echo sequence produced a gapless series of thin transverse sections (TE: 9.2 msec; TR: 21 msec; flip angle: 30°; field of view: 256 mm; acquisition matrix: 256×256; slice thickness: 1 mm).

### Regions of interest

All MR images were coregistered to the PET images with the statistical parametric mapping (SPM2) system [35]. Regions of interest (ROIs) were drawn manually on coregistered MR images and transferred to the PET images. ROIs were defined for the cerebellar cortex, putamen, caudate head, and occipital cortex (Fig. 2). Each ROI was drawn on three adjacent sections and data were pooled to obtain the average radioactivity concentration for the whole volume of interest. To obtain regional time-activity curves, regional radioactivity was calculated for each frame, corrected for decay, and plotted versus time. ROIs were drawn by in-house software. No software correction for head movement during PET measurements was applied to the dynamic PET images.

### Calculation of occupancy of dopamine D_2_ receptors

For PET studies with [^11^C]raclopride, the binding potential (BP_ND_) was calculated by the reference tissue model method [36], [37], with which the time-activity curve in the brain region is described by that in the reference region with no specific binding, assuming that both regions have the same level of nondisplaceable radioligand binding:
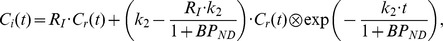
where C_i_ is the radioactivity concentration in a brain region; C_r_ is the radioactivity concentration in the reference region; R_I_ is the ratio of K_1_/K_1_′ (K_1_, influx rate constant for the brain region; K_1_′, influx rate constant for the reference region); k_2_ is the efflux rate constant for the brain region; 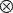 denotes the convolution integral. In this analysis, three parameters (BP_ND_, R_I_, and k_2_) were estimated by non-linear least-squares curve fitting. The cerebellum was used as reference region. Dopamine D_2_ receptor occupancy by aripiprazole was calculated as follows:
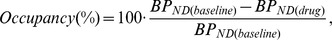
where BP_ND(baseline)_ is the BP_ND_ value in the baseline study, and BP_ND(drug)_ is the BP_ND_ value in the drug challenge study.

### Calculation of dopamine synthesis capacity

The uptake rate constant for L-[β-^11^C]DOPA, indicating the dopamine synthesis capacity, was estimated by graphical analysis [38]–[40], which allows for calculation of the uptake rate constant k_i_ using time-activity data in a reference brain region with no irreversible binding. The k_i_ values can be estimated by simple linear least-squares fitting as follows:
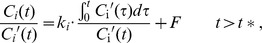
where C_i_ and C_i_′ are the total radioactivity concentrations in a brain region with and without irreversible binding, respectively, and t* is the equilibrium time of the compartment for unchanged radiotracer in brain tissue. Plotting 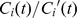 versus 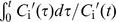, after time t*, yields a straight line with the slope k_i_ and intercept F. In the present study, the occipital cortex was used as reference region with no irreversible binding, because this region is known to have the lowest dopamine concentration [41] and least AADC activity [42]. The equilibrium time t* was set to be 29 min, and data plots of 29 to 89 min were used for linear least-squares fitting [32], [43]. The percentage change in k_i_ by oral administration of aripiprazole was calculated as follows:
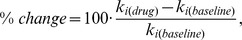
where k_i(baseline)_ is the k_i_ value in the baseline study, and k_i(drug)_ is the k_i_ value in the drug challenge study.
